# Supplementary material for: Genome-Wide Comparative Analysis of Lactiplantibacillus pentosus Isolates Autochthonous to Cucumber Fermentation Reveals Subclades of Divergent Ancestry
Source: Foods. 2023 Jun 23;12(13):2455. doi: 10.3390/foods12132455 (PMC10340309; doi:10.3390/foods12132455)

**Supplementary Figure S1.** The putative protospacer adjacent motifs (PAMs) were predicted for Type II-A (A) and Type I-E (B) CRISPR-Cas systems present in *L. plantarum* and *L. pentosus* genomes, using protospacer flanking sequences and the consensus sequences illustrated at the top.

A)

Consensus

Identity

*L. plantarum* 3.2.8 – CRISPR 1 – spacer 4

*L. plantarum* 7.8.4 – CRISPR 1 – spacer 6

*L. pentosus* 3.2.37 – CRISPR 2 – spacer 8

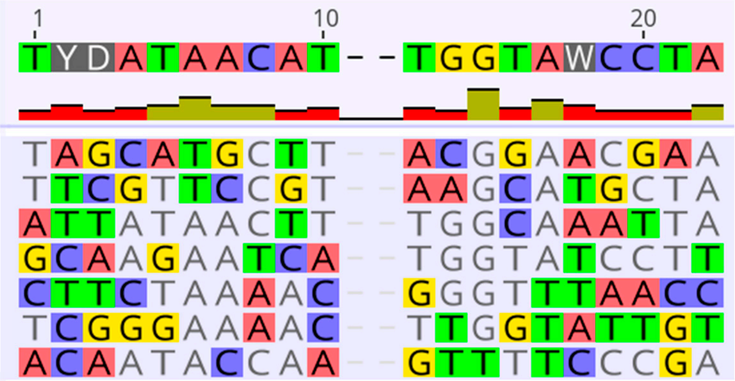


B)

Consensus

Identity

*L. pentosus* 7.2.11 – CRISPR 3 – spacer 18

*L. pentosus* 7.2.11 – CRISPR 3 – spacer 20

*L. pentosus* 7.2.4 – CRISPR 2 – spacer 7

*L. pentosus* 7.2.11 – CRISPR 2 – spacer 3

*L. pentosus* 7.2.11 – CRISPR 3 – spacer 30

*L. pentosus* 7.2.4 – CRISPR 1 – spacer 1

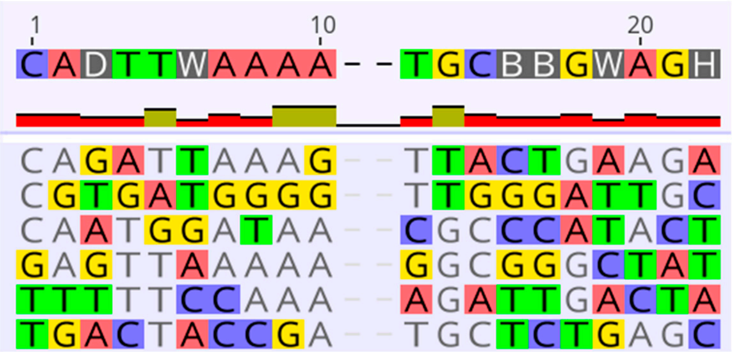

Supplement: Supplementary file 1 [file foods-12-02455-s001.zip › SM Figure S1.pdf]
